# Expression of *Camelina* WRINKLED1 Isoforms Rescue the Seed Phenotype of the *Arabidopsis wri1* Mutant and Increase the Triacylglycerol Content in Tobacco Leaves

**DOI:** 10.3389/fpls.2017.00034

**Published:** 2017-01-24

**Authors:** Dahee An, Hyojin Kim, Seulgi Ju, Young Sam Go, Hyun Uk Kim, Mi Chung Suh

**Affiliations:** ^1^Department of Bioenergy Science and Technology, Chonnam National UniversityGwangju, South Korea; ^2^Department of Bioindustry and Bioresource Engineering, Plant Engineering Research Institute, Sejong UniversitySeoul, South Korea

**Keywords:** *Camelina sativa*, fatty acid, leaves, oil, triacylglycerol, WRINKLED1

## Abstract

Triacylglycerol (TAG) is an energy-rich reserve in plant seeds that is composed of glycerol esters with three fatty acids. Since TAG can be used as a feedstock for the production of biofuels and bio-chemicals, producing TAGs in vegetative tissue is an alternative way of meeting the increasing demand for its usage. The *WRINKLED1* (*WRI1*) gene is a well-established key transcriptional regulator involved in the upregulation of fatty acid biosynthesis in developing seeds. WRI1s from *Arabidopsis* and several other crops have been previously employed for increasing TAGs in seed and vegetative tissues. In the present study, we first identified three functional *CsWRI1* genes (*CsWRI1A. B*, and *C*) from the *Camelina* oil crop and tested their ability to induce TAG synthesis in leaves. The amino acid sequences of *CsWRI1s* exhibited more than 90% identity with those of *Arabidopsis WRI1*. The transcript levels of the three *CsWRI1* genes showed higher expression levels in developing seeds than in vegetative and floral tissues. When the *CsWRI1A. B*, or *C* was introduced into *Arabidopsis wri1-3* loss-of-function mutant, the fatty acid content was restored to near wild-type levels and percentages of the wrinkled seeds were remarkably reduced in the transgenic lines relative to *wri1-3* mutant line. In addition, the fluorescent signals of the enhanced yellow fluorescent protein (eYFP) fused to the *CsWRI1* genes were observed in the nuclei of *Nicotiana benthamiana* leaf epidermal cells. Nile red staining indicated that the transient expression of *CsWRI1A. B*, or *C* caused an enhanced accumulation of oil bodies in *N. benthamiana* leaves. The levels of TAGs was higher by approximately 2.5- to 4.0-fold in *N. benthamiana* fresh leaves expressing *CsWRI1* genes than in the control leaves. These results suggest that the three *Camelina* WRI1s can be used as key transcriptional regulators to increase fatty acids in biomass.

## Introduction

*Camelina sativa* L. is an underdeveloped oil crop in the family Brassicaceae, which has several advantages in the agronomic and environmental context over the current developed oil crops. *Camelina* has a relatively short growing period (85–100 days to maturity) and can be cultivated twice in 1 year (Putnam et al., 1993). In comparison with other oil crops, it requires lower amounts of fertilizer for growth and is more resistant to various stresses such as cold and drought (Putnam et al., 1993; Kim et al., 2013; Bansal and Durrett, 2016). *Camelina* seed oil is composed of 35–45% triacylglycerol (TAG), which has a high proportion of polyunsaturated fatty acids (PUFAs) (Lu and Kang, 2008; Bansal and Durrett, 2016). Approximately 40% of the total fatty acid (FA) content is composed of linolenic acid (18:3) and linoleic acid (18:2). The considerable amount of PUFAs confers considerable susceptibility to oxidation, which makes it less suitable for biodiesel production and domestic cooking, such as frying vegetables (Soriano and Narani, 2012). However, breeding and genetic engineering programs can generate new varieties of *Camelina* with a lower PUFA content for stable oxidation (Kang et al., 2011; Nguyen et al., 2013). *Camelina* can be transformed using the *Agrobacterium*-mediated flower-dip method, which is a relatively simple and rapid route to generating transgenic plants with superior agronomic traits (Lu and Kang, 2008; Liu et al., 2012). The whole genome sequence and seed transcriptome data present valuable resources for the understanding of the function of genes involved in oil biosynthesis in *Camelina* seeds (Hutcheon et al., 2010; Nguyen et al., 2013; Kagale et al., 2014).

Triacylglycerol is a neutral lipid molecule that stores carbons and hydrogens that are utilized for energy production in the life cycle of plants (Athenstaedt and Daum, 2006). TAGs are mainly observed in seeds, where they are used as energy stores for seed germination; they also occur in chloroplasts of senescent leaves where they aid in fatty acid sequestration and in pollen, where they have been shown to promote pollen germination in *Arabidopsis* (Kaup et al., 2002; Kim et al., 2002; Athenstaedt and Daum, 2006). The first step of fatty acid synthesis in seeds is the formation of malonyl-coenzyme A (CoA) from acetyl-CoA by acetyl-CoA carboxylase (ACCase). Malonyl-CoA-ACP malonyltransferase catalyzes the conversion of malonyl-CoA to malonyl-ACP. Then, malonyl-ACP is condensed with acetyl-CoA to make 3-ketoacyl-ACP. After that the series of sequential condensation reactions of malonyl-ACP to 3-ketoacyl-ACP or the growing of acyl-ACP by the fatty acid synthase complex results in 16- to 18-carbon fatty acyl-ACPs in the plastids (Chapman and Ohlrogge, 2012). The fatty acyl group is hydrolyzed by thioesterases (FATA/FATB). The fatty acyl chains are exported to the cytoplasm and activated to fatty acyl-CoAs by long-chain acyl-CoA synthetase (LACS). Fatty acyl-CoA pools are utilized for the esterification of fatty acids with glycerol-3-phosphate (G3P) sequentially by three acyltransferases called G3P acyltransferase (GPAT), lysophosphatidic acid acyltransferase (LPAT), and diacylglycerol acyltransferase (DGAT) in the endoplasmic reticulum (ER) to synthesize TAG. Oil bodies containing TAG are formed in the ER membrane budding from the TAG synthesizing site as a droplet (Li-Beisson et al., 2010).

The network for AFL master regulators, ABSCISIC ACID INSENSITIVE3 (ABI3), FUSCA3 (FUS3), LEAFY COTYLEDON1 (LEC1), and LEC2 have been studied with the aim of controlling the biosynthesis of *Arabidopsis* seed oil (North et al., 2010; Roscoe et al., 2015; Devic and Roscoe, 2016). This network can regulate the expression of genes encoding enzymes that synthesize the storage lipids and protein reserves in seeds. As a key transcription factor for lipid accumulation, WRINKLED1 (WRI1) is located downstream in this AFL network (Roscoe et al., 2015). Ectopic expression of *FUS3* can regulate *WRI1* expression (Yamamoto et al., 2010). LEC2 may regulate fatty acid biosynthesis genes through direct regulation of WRI1 (Baud et al., 2007). Although ABI3 is not known to directly regulate the expression of *WRI1*, it is known that ABI3, FUS3, LEC1, and LEC2 regulate the downstream genes through synergistic interactions with each other during seed maturation (Devic and Roscoe, 2016). The *WRI1* gene was first identified in an *Arabidopsis* mutant exhibiting wrinkled seed morphology; it encodes an AP2/EREBP type transcription factor (Focks and Benning, 1998). WRI1 regulates the expression of genes encoding the enzymes involved in catalytic processes such as phosphoglycerate mutase, plastidic pyruvate kinase β subunit 1 (PI-PKβ1), and pyruvate dehydrogenase (PDHE1α) enzymes during glycolysis, wherein it has been observed to increase the amount of pyruvate during acetyl-CoA synthesis (Baud et al., 2007, 2009; Maeo et al., 2009; Fukuda et al., 2013). Furthermore, WRI1 directly binds to the promoters of genes encoding key enzymes for fatty acid biosynthesis during seed maturation, such as biotin carboxyl carrier protein isoform 2 (BCCP2), acyl carrier protein 1 (ACP1), and keto-ACP synthase 1 (KAS1) (Baud et al., 2009; Maeo et al., 2009).

*Arabidopsis wri1* mutants have been shown to exhibit an up to 80% lower TAG content than the wild-type seeds, with correspondingly increased sucrose levels. This implies that *WRI1* is a regulator for carbon allocation between sucrose and fatty acids in developing seeds (Focks and Benning, 1998; Ma et al., 2015). Following the discovery of *Arabidopsis WRI1* (*AtWRI1*), its homologues have been identified from the seeds of rapeseed (*Brassica napus* L.), corn (*Zea mays* L.), and oil palm (*Elaeis guineensis* Jacq.) (Liu et al., 2010; Shen et al., 2010; Pouvreau et al., 2011; Ma et al., 2013; Wu et al., 2014). In addition to seeds, *WRI1* homologs have been identified in non-seed tissues containing oil, such as the stem of poplar (*Populus trichocarpa* L.), tuber parenchyma of nutsedge (*Cyperus esculentus* L.), and leaf blades of *Brachypodium distachyon* L. Beauv. (Grimberg et al., 2015; Yang et al., 2015). Overexpression of the *WRI1* genes contributed to increased TAG levels in the seeds and vegetative tissues. Seed-specific expression of *AtWRI1* transgene increased TAG content to levels 14–30% higher than that in the wild-type seeds in *Arabidopsis. B. napus*, corn, and *Camelina* (Cernac and Benning, 2004; Liu et al., 2010; Shen et al., 2010; Pouvreau et al., 2011; Wu et al., 2014; An and Suh, 2015). Ectopic overexpression of *WRI1* stimulated oil production in the vegetative tissues (Vanhercke et al., 2013, 2014; Nookaraju et al., 2014; Zale et al., 2016). Five *WRI1* isoforms have been isolated from diverse species such as *Arabidopsis*, potato (*Solanum tuberosum* L.), oat (*Avena sativa* L.), and nutsedge. These genes were introduced into leaves using the agroinfiltration method, which resulted in an increase in TAG content from 0.05% to 2.20% in *Nicotiana benthamiana* Domin. leaves (Grimberg et al., 2015).

In the present study, we first identified three *Camelina WRI1* isoforms, *CsWRI1A, CsWRI1B*, and *CsWRI1C*, that complemented the wrinkled-seed phenotype and partially restored TAG content in *Arabidopsis wri1-3* mutants. We then studied the *CsWRI1* genes to identify a potential candidate to act as a transcriptional regulator for inducing TAG production in vegetative tissues.

## Materials and Methods

### Plant Materials and Growth Conditions

*Camelina sativa* L. CAME, *Arabidopsis thaliana* accession Columbia-0, and *N. benthamiana* plants were grown in long-day growth conditions with 16 h/8 h light/dark photoperiod at 21–24°C, and 50–60% humidity in a sterilized soil mixture (peat moss enriched soil:vermiculite:perlite in 4:2:1 ratio) (An and Suh, 2015).

### Gene Identification and Isolation

Total RNAs were isolated from developing *Camelina* seeds 20 days after flowering (DAF) based on the protocol for RNA isolation from seeds (Oñate-Sánchez and Vicente-Carbajosa, 2008). cDNAs were then synthesized by RT-PCR using primers listed in **Supplementary Table 1**. The PCR protocol was as follows: denaturation at 94°C for 30 s, annealing at 58–62°C for 20 s, and extension at 72°C for 120 s. PCR products were cloned into the pGEM T-easy vector (Promega) and their nucleotide sequences were determined.

### Protein Sequence and Phylogenetic Tree Analysis

Amino acid sequences were aligned using CLUSTALW, and dendrograms for phylogenetic analysis were constructed using the MEGA 6.06 program^1^ with the maximum likelihood method and a bootstrap value of 500 replicates (Tamura et al., 2013).

### RT-PCR Analysis

In *Camelina*, transcript expression was analyzed in various tissues of the aerial parts such as the flower buds and leaves of 5-week-old plants, stems from 4-week-old plants, roots from 2-week-old plants, open flowers from plants older than 6 weeks, and developing seeds 10, 20, and 30 DAF. To assess the expression level of the transgene in *Arabidopsis* complementation lines, total RNAs were extracted from developing seeds of transgenic *Arabidopsis* 6–8 DAF. Total RNAs were isolated from these tissues using QIAGEN RNeasy^®^ plant mini kit (50), following the manufacturers protocols. In addition, RNAs from developing seeds of *Camelina* and *Arabidopsis* were isolated according to the method described in Oñate-Sánchez and Vicente-Carbajosa (2008). First, cDNAs were synthesized from these RNAs by the GoScript^TM^ reverse transcription system (Promega) followed by PCR by the Access Quick^TM^ RT-PCR system (Promega) to analyze the transcript levels of each gene using gene-specific primers (**Supplementary Table 1**). The PCR protocol was as follows: denaturation at 94°C for 30 s, annealing at 58–62°C for 20 s, and extension at 72°C for 20 s. These cycles were performed 35 times. To control equal cDNA loading in RT-PCR, *CsACTIN11* and *EIF4A1*(*At3g13920*) gene-specific primers listed in **Supplementary Table 1** were used as controls for transcript levels in various tissues (Hutcheon et al., 2010).

### Vector Construction

To express the *CsWRI1* gene in transgenic plants, each cDNA containing an ORF was inserted under the sequences of CaMV 35S promoter by digestion at the *Bam*HI (5′-terminus) and *Sac*1 (3′-terminus) restriction endonuclease sites in the pBA002 vector (Kim et al., 2006). This vector contains the herbicide resistance marker gene *PAT* (Phosphinothricin acetyltransferase), which enables selection of the transgenic plants. These constructs were transformed into *Agrobacterium tumefaciens* GV3101 using the freeze-thaw method (An, 1987), which were then used for the transformation of *Arabidopsis* by the *Agrobacterium*-mediated floral dip method (Zhang et al., 2006).

*CsWRI1* cDNAs containing an ORF without the stop codon were inserted at the *Sac*I (5′-terminus) and *Xma*I (3′-terminus) sites between the CaMV 35S promoter and enhanced yellow fluorescent protein (eYFP) to enable fusion of the eYFP with the in-frame stop codon, and allow expression under the control of the CaMV 35S promoter in the pPZP212 vector (GenBank U10462). These constructs were then used for the detection of fluorescence signals of CsWRI1 proteins in the epidermal cells of *N. benthamiana* infected by *Agrobacterium tumefaciens* GV3101.

### Subcellular Localization

The vectors expressing *CsWRI1s:eYFP* under the control of the CaMV 35S promoter were transformed into *Agrobacterium tumefaciens* LBA4404, followed by the infection of the *N. benthamiana* leaf epidermis using the agroinfiltration method (Yoo et al., 2007). The *N. benthamiana* leaves were further grown for 12 h, and the fluorescence was observed using the TCS SP5 AOBS/Tandem laser scanning confocal microscope (Leica Microsystems, Germany). The emission wavelength was 571–617 nm and the excitation wavelengths were 520–554 nm for YFP and 572–618 nm for RFP.

### Generation of Transgenic *Arabidopsis*

Progeny seeds harvested from the transformed *Arabidopsis* plants were germinated and grown on 1/2 MS medium (1% sucrose, 0.7% phytoagar) containing 5 μg/mL of phosphinothricin (PPT). PPT-resistant T_1_ plants were tested for the presence of the transgene in the genome by PCR analysis with primers listed in **Supplementary Table 1**. T_2_ seeds from T_1_ transgenic plants were selected for the observation of seed morphology and determination of the fatty acid content.

### Seed Morphology Analysis

Seed morphology was observed using light and scanning electron microscopes. Dry seeds of the wild-type and the *wri1-3* mutant were coated with platinum particles using a Hitachi E1030 coater on aluminum stubs. Their images were scanned using a field emission scanning electron microscope (FE-SEM, Hitachi S-4700, Tokyo, Japan). The ratio of seeds rescued from the wrinkled phenotype to normal in the transgenic lines was recorded from the photographs taken of the T_2_ seed siblings using an Axiocam, MRc 5 camera equipped with a light microscope (Carl Zeiss: SteREO Lumar. V12).

### Nile Red Staining

The leaves of *N. benthamiana* were infiltrated with *Agrobacterium* containing an empty vector (pBA002) or the *CsWRI1* vector. These leaves were stained with Nile red solution [10 μg/mL in 0.1 M Tris-HCl buffer (pH 8) Sigma-N3013] at room temperature for 30 min. The stained leaves were washed with 0.1 M Tris-HCl buffer (pH 8) for 10 min, followed by observation of red fluorescence of the oil bodies at wavelengths of 560 nm for excitation and 615 nm for emission using a tandem laser confocal scanning microscope (TCS SP5 AOBS, Leica Microsystems, Germany).

### Thin Layer Chromatograph (TLC) Analysis

The fresh leaves of *N. benthamiana* were homogenized and immersed in isopropanol at 65°C for 15 min. Chloroform and water were mixed in same tube, then total lipids were extracted twice using a chloroform:methanol solution (2:1, v/v). The solvents of the lipid phase washed with 1 M KCl were collected and evaporated using nitrogen gas. Finally, total lipids were dissolved in chloroform containing tri 17:0-TAG (glyceryl triheptadecanoate; Sigma-T2151) as an internal standard and separated by TLC (Silica gel 60, MERCK) in hexane:diethylether:acetic acid (70:30:1, v/v/v). They were then visualized by spraying 0.01% primuline (Sigma-206865) in 80% acetone under UV light (Vanhercke et al., 2013). TAG bands scraped from TLC were dissolved in toluene and esterified with methanol and H_2_SO_4_ mixture at 95°C for 90 min. The esterified TAGs were analyzed for their fatty acid content using gas chromatography as described in the following section.

### Fatty Acid Analysis

To measure total fatty acid profile and content, total lipids extracted from *Arabidopsis* seeds or *N. benthamiana* fresh leaves and tri 17:0-TAG in toluene were combined and esterified with methanol mixed with H_2_SO_4_ at 95°C for 90 min. After the reaction, 0.9% NaCl solution and hexane were added to extract the fatty acid methyl esters. The upper phase containing the fatty acid methyl esters was concentrated by N_2_ air and separated in a DB23 column (30 m × 0.25 mm, 0.25 μm film thickness; J&W Scientific, Folsom. CA, USA) using GC-2010 gas chromatography (Shimadzu, Japan). The temperature of the reaction ranged from 160°C to 220°C, increasing at a rate of 2.5°C per min. The peak area on the retention time for each fatty acid was characterized and measured by comparison with the known standard fatty acid profiles, and the concentration of each fatty acid was calculated by comparing its peak area with that of the internal standard.

## Results

### *Camelina* Possesses Three Copies of *WRI1*

**FIGURE 1:**
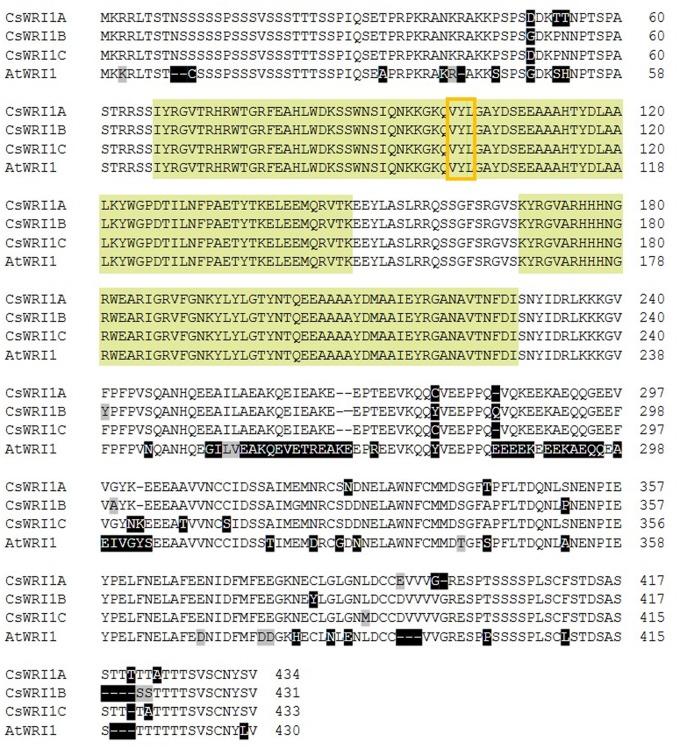
**Alignment of the deduced amino acid sequences of *WRI1* isoforms from *C. sativa* and *A. thaliana*.** Non-conserved and conservatively changed amino acid residues are shaded in black and gray, respectively. Bright green and orange boxes indicate conserved AP2/EREBP DNA binding motifs and the ‘VYL’ motif for transcriptional activation of WRI1, respectively.

In order to identify the factors involved in the accumulation of higher oil content in *Camelina*, we first focused on identifying the homologous genes of *Arabidopsis WRINKLED1* (*AtWRI1*) in two different *Camelina* genome databases^2^^,^^3^. Three *WRI1* scaffolds and three *WRI1* loci were identified in different chromosomes of the hexaploid genome (**Supplementary Table 2**) (Kagale et al., 2014). Three *WRI1* scaffolds showed overall 28–36% nucleotide sequence identity in intron and 5′- and 3′-nocoding regions (**Supplementary Figure 1**) (Kagale et al., 2014). Based on the differences in nucleotide sequences we can detect the corresponding three distinct *WRI1* transcripts from developing seeds in *Camelina* by cloning their cDNAs. These three *Camelina* cDNAs were designated as *CsWRI1A* (GenBank KY129795), *CsWRI1B* (GenBank KY129796), and *CsWRI1C* (GenBank KY129797) based on the identity with *WRI1* genomic DNA sequences in *Camelina* genome scaffolds and *Arabidopsis WRI1*. Excluding the non-coding regions and the intron, the three *CsWRI1* genes exhibited a high identity in nucleotide sequences (**Supplementary Figure 2**). Compared with that of the AtWRI1, the three CsWRI1s exhibited a high identity in the amino acid (aa) sequence levels (∼95%) with 4, 1, and 3 amino acids that are longer than that of the 430 aa open reading frame (ORF) of *Arabidopsis*. An AP2/EREBP DNA binding motif and a VYL transcriptional activation motif are conserved between *Camelina* and *Arabidopsis* (**Figure 1**).

**FIGURE 2:**
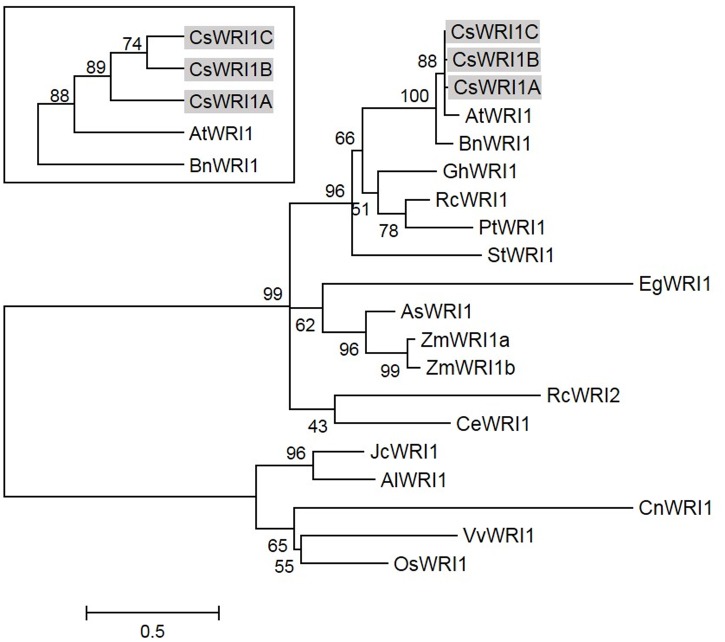
**Phylogenetic tree of WRI1 from higher plants including *C. sativa*.** Phylogenetic tree was generated using MEGA6.06 by the maximum likelihood method. Bootstrap value percentages of 500 replicates are shown at the branching points. AlWRI1, *Arabidopsis lyrata* (EFH52510.1); AsWRI1, *Avena sativa* (SRX1079426); BnWRI1, *Brassica napus* (ABD16282.1); CeWRI1, *Cyperus esculentus* (SRX1079431); CnWRI1, *Cocos nucifera* (JQ040545); EgWRI1, *Elaeis guineensis*; GhWRI1, *Gossypium hirsutum* (TC200263); JcWRI1, *Jatropha curcas* (AIA57945.1); OsWRI1, *Oryza sativa* (CAE00853.1); PtWRI1, *Populus trichocarpa* (SRX1079428); RcWRI1, *Ricinus communis* (AB774159.1, AB774160.1); StWRI1, *Solanum tuberosum* (SRX1079426); VvWRI1, *Vitis vinifera* (CBI32013.3); ZmWRI1, *Zea mays* (ACF83189.1, ACF80269.1).

Several *WRI1* genes were identified from crop plants following *Arabidopsis* (**Figure 2**) (Shen et al., 2010; Wu et al., 2014). The majority are single *WRI1* genes identified from each species, except for maize, in which two isoforms, *ZmWRI1a* and *ZmWRI1b*, are present in the genome. Owing to its hexaploid genome, *Camelina* has three WRI1 isoforms, which is the highest number of copies discovered in any plant species to date. Phylogenetic analysis indicates that the CsWRI1s are grouped in the same clade as these of *Arabidopsis* and *B. napus* among the various WRI1s that are currently identified from plants (**Figure 2**).

### Three *WRI1s* Are Predominantly Expressed in Developing Seeds

**FIGURE 3:**
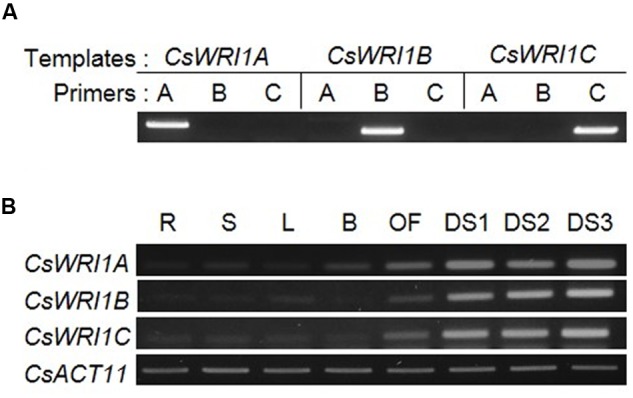
**Expression of three *CsWRI1* isoforms in various *C. sativa* organs.** **(A)** The primer specificity of three *CsWRI1* isoforms. Plasmids harboring *CsWRI1A. CsWRI1B*, or *CsWRI1C* were used in PCR analysis as templates. PCR products were analyzed on 1% agarose gels. **(B)** Total RNAs were isolated from the roots (R) of 2-week-old stems (S) of 4-week-old, leaves (L) and buds (B) from 5-week-old, and open flowers (OF) and developing seeds (DS1, DS2, and DS3; 10, 20, and 30 days after flowering, respectively) of 6-week-old *C. sativa* plants.

Since *Arabidopsis WRI1* is known to express during seed development (Baud et al., 2007), the three *CsWRI1s* were analyzed for the expression pattern of their transcripts in developing seeds, which was then compared with that of the various tissues in *Camelina*. To distinguish the transcript of each *CsWRI1* isoform from the three highly homologous genes, transcript-specific primers were designed and their specificity was confirmed by cDNA-PCR. **Figure 3A** shows each set of primers that were able to detect the specific transcript for *CsWRI1* isoform. Using these gene-specific primers, RT-PCR analysis were performed in samples of the root, stems, leaves, flower buds, open flowers and three different stages of developing seeds. The expression levels of *CsWRI1A, CsWRI1B*, and *CsWRI1C* were predominantly higher in developing seeds than in other organs (**Figure 3B**). The Csa06g028810 transcript, showing 98% identity with *CsWRI1B*, represents high expression in developing seeds in the transcriptome analysis (**Supplementary Table 3**). These results indicate that all three *CsWRI1* isoforms are actively transcribed during seed development.

### Three WRI1 Are Localized in the Nucleus

**FIGURE 4:**
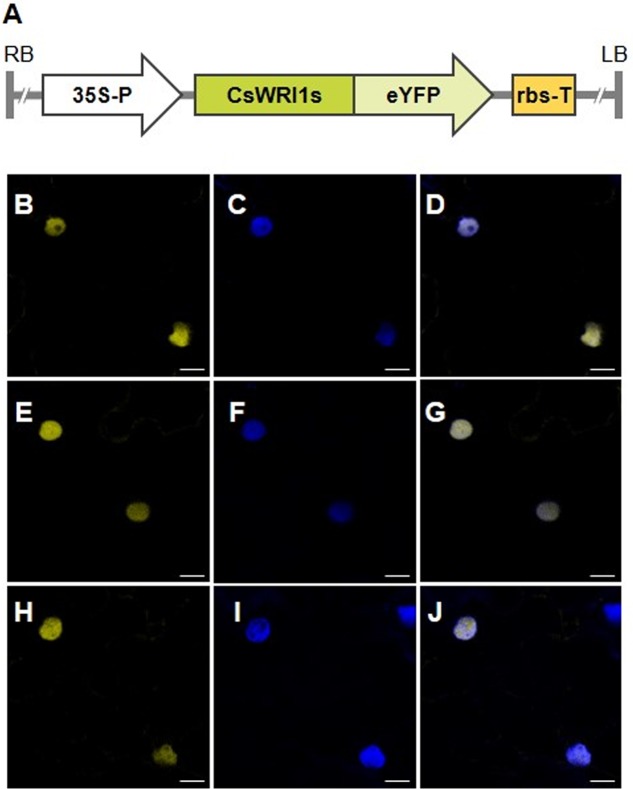
**Subcellular localization of CsWRI1s:eYFP proteins in *N. benthamiana* epidermis.** **(A)** Schematic diagram of *CsWRI1A:eYFP. CsWRI1B:eYFP*, and *CsWRI1C:eYFP* constructs. 35S-P, cauliflower mosaic virus 35S promoter; LB, left border; RB, right border; rbs-T, the terminator of ribulose-1,5-bisphosphate carboxylase and oxygenase small subunit from pea (*Pisum sativum)*. **(B–J)** *Agrobacterium* harboring the CsWRI1A:eYFP (upper row), CsWRI1B:eYFP (middle row) or CsWRI1C:eYFP (bottom row) construct was infiltrated into *N. benthamiana* leaves and then the fluorescent signals were visualized under laser confocal scanning microscopy. YFP signals **(B,E,H)** from the CsWRI1s:eYFP constructs. The nucleus **(C,F,I)** was visualized by staining with DAPI under the UV filter. Merged image between signals of YFP and the nucleus **(D,G,J)**. EV, empty vector (pBA002). Bars = 10 μm.

The amino acid sequences derived from the three *CsWRI1* cDNAs revealed that the protein might be a transcription factor containing the AP2/EREBP DNA binding and VYL activation motifs (**Figure 1**). In order to detect the subcellular localization of *Camelina* WRI1, each *CsWRI1* cDNA was fused in-frame with eYFP under the CaMV 35S promoter in the *Agrobacterium* binary expression vector (**Figure 4A**). Agrobacteria containing the *CsWRI1s:eYFP* genes were infiltrated into *N. benthamiana* leaves and the fluorescent signals were visualized under the confocal scanning microscope. The yellow fluorescent signals emitted by CsWRI1:eYFP were detected throughout the nucleus, except for the nucleolus, in all three WRI1 isoforms (**Figures 4B,E,H**). These signals identically overlapped with the blue fluorescent signal (**Figures 4C,F,I**) emitted by the nuclei stained with 4′,6-diamidino-2-phenylindole (DAPI) under UV light. The subcellular localization of CsWRI1A, CsWRI1B, and CsWRI1C indicate that all three isoforms localize in the nucleus (**Figures 4D,G,J**).

### Three CsWRI1s Functionally Rescued the *Arabidopsis wri1-3* Mutant Phenotype

**FIGURE 5:**
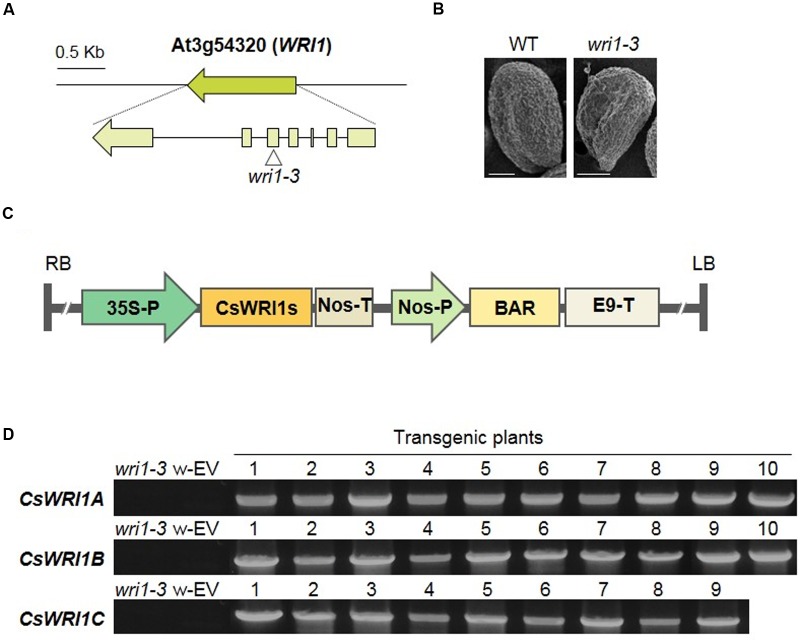
**Isolation of T-DNA inserted *wri1-3* knockout mutant and complementation of *CsWRI1A. CsWRI1B*, and *CsWRI1C* in *wri1-3* mutants.** **(A)** Genomic organization of the *WRI1* gene inserted with T-DNA in *wri1-3*. **(B)** SEM image of the seed morphology of WT and *wri1-3* mutant seeds. Bar = 10 μm. **(C)** Schematic diagram of the binary vector constructs for the expression of *CsWRI1*s in *Arabidopsis wri1-3* mutant. **(D)** Genomic DNA-PCR of *CsWRI1s* transgenes of WT, *wri1-3* mutant, and complementation transgenic lines. w-EV represents transgenic *Arabidopsis wri1-3* mutant introducing empty vector. The numbers indicate independent transgenic lines (T_1_).

*Arabidopsis* has a single *WRI1* gene in its genome. Mutant seeds with T-DNA inserted in the fifth exon of *AtWRI1* were obtained from the *Arabidopsis* Biological Resource Center (ABRC) and we identified the *wri1-3* homozygous mutant (**Figure 5A**). This mutant exhibited the wrinkled phenotype in seeds, in contrast to the round phenotype of wild-type seeds (**Figure 5B**). To test if the three *Camelina WRI1* genes can work functionally instead of *AtWRI1* in the *wri1-3* mutant, each of the *CsWRI1* cDNAs was inserted between the CaMV 35S promoter and *nos* terminator in plant expression vectors (**Figure 5C**). These vectors were transformed into *wri1-3*, and the generated transgenic plants were selected based on the resistance to the herbicide Basta. Among the Basta resistant transgenic plants, 9–10 transgenic plants were analyzed for the presence of the transgene insert by the genomic DNA-PCR method with gene-specific primers for each *CsWRI1*. Transgenes were detected in genome DNA isolated from the leaves of T_1_ transformants, but not in that of *wri1-3* and empty vector transgenic leaves (**Figure 5D**).

**FIGURE 6:**
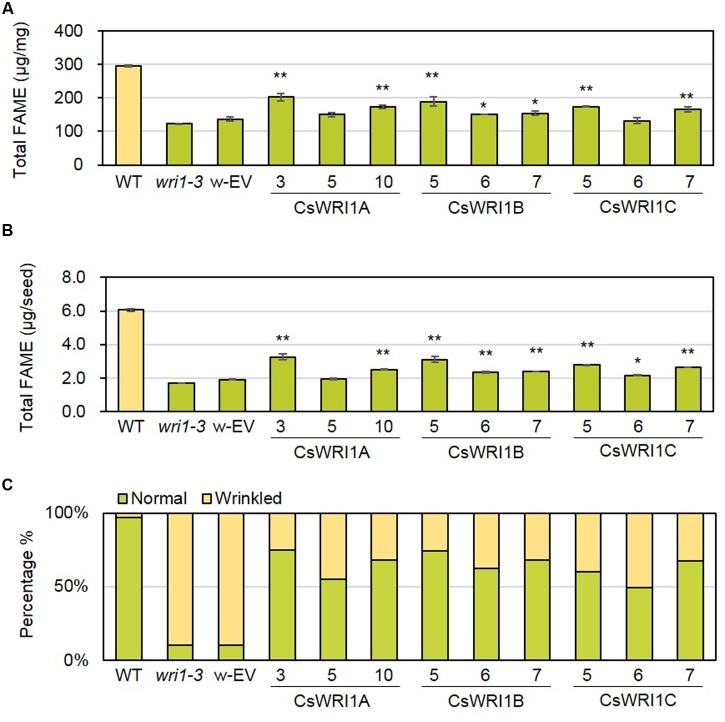
**Fatty acid content and rescue of the wrinkled phenotype from seeds of WT, *wri1-3*, and transgenic plants.** Fatty acids were extracted from dry seeds of WT, *wri1-3*, and transgenic plants (T_2_), transmethylated, and analyzed using gas chromatography. Fatty acid content was expressed on the basis of seed dry weight **(A)** or seed number **(B)**. Error bar indicates SE of three independent measurements. (^∗∗^*P* < 0.01; ^∗^*P* < 0.05; Student’s *t*-test). **(C)** The graph showing morphology of dry seeds from WT, *wri1-3*, and transgenic lines (T_2_). w-EV represents transgenic *Arabidopsis* of *wri1-3* mutant introducing empty vector. The numbers indicate independent transgenic lines.

In the transgenic *Arabidopsis wri1-3* plants expressing *CsWRI1s*, the fatty acid content of seeds in the T_2_ generation in nine to ten independent lines for each *Camelina WRI1* isoform was measured and compared with those of the *wri1-3*, empty vector transformed *wri1-3*, and wild-type plants (**Supplementary Table 4**, **Figure 6**). To detect the fatty acid amount in seeds accurately, the dry weight of seeds and seed number were used for the measurement in three representative lines for each transgene. All three *CsWRI1* cDNAs were able to partially complement the *wri1-3* phenotype, as evidenced by a greater seed fatty acid content compared to the mutant and the vector-only transformant control irrespective of expression on the basis of seed weight or seed number (**Figures 6A–C**, **Supplementary Table 5**). These results suggest that CsWRI1A, B, and C variants are each potentially involved in regulating the level of fatty acid biosynthesis in developing seeds.

**FIGURE 7:**
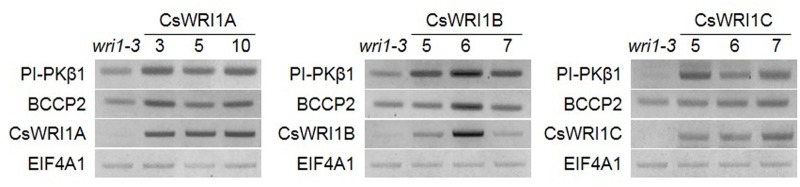
**Expression of *CsWRI1*s and WRI1 downstream targets in developing *Arabidopsis* seeds.** Total RNA was isolated from developing seeds 6–8 days after flowering of *wri1-3* mutant and transgenic plants (T_2_). The isolated RNAs were subjected to RT-PCR analysis. The *EIF4A1* gene was used to determine RNA quality and quantity. PI-PKβ1, At5g52920. BCCP2, At5g15530.

We analyzed transcript levels of each *CsWRI1* transgene in developing seeds with three best rescued plants for each *CsWRI1* gene among all of the transgenic lines and compared them with the *wri1-3* host line. RT-PCR analysis showed that each *CsWRI1A. B*, and *C* were expressed in developing seeds of transgenic plants and enhanced the expression level of genes involved in glycolysis and fatty acid synthesis, *PI-PKβ1* and *BCCP*2 (**Figure 7**). These RT-PCR results showed correlation between the expression of *CsWRI1* transgene, the partial complementation of seed fatty acid content, and seed morphology.

### Ectopic Expression of Three *CsWRI1*s Induces Accumulation of Oil Bodies and Increases Fatty Acid Content in *N. benthamiana* Leaves

**FIGURE 8:**
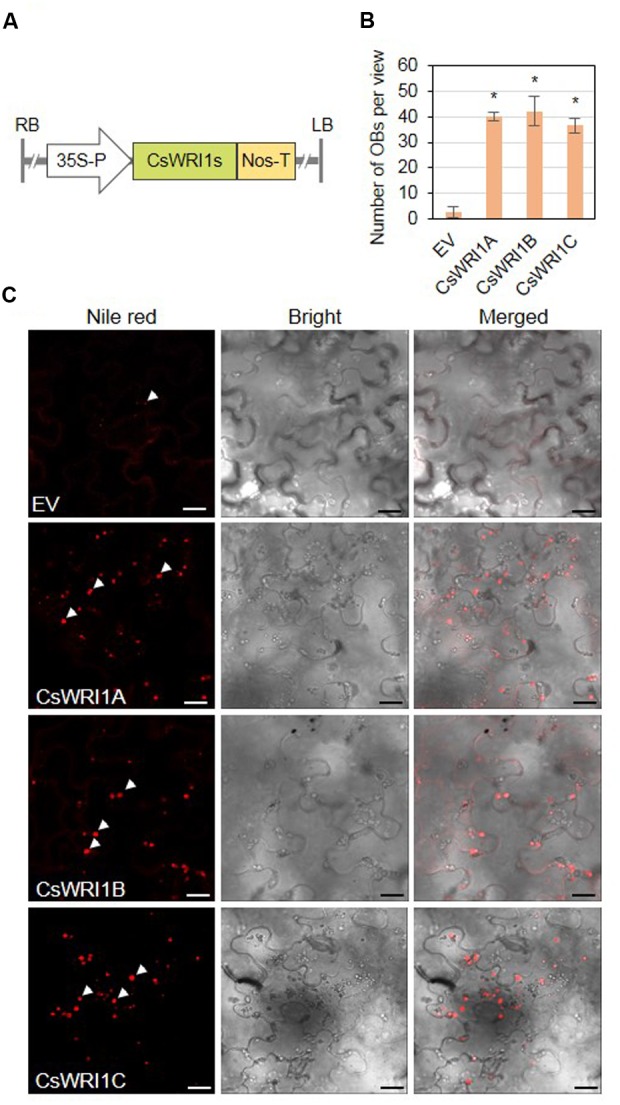
**Transient expression of three *CsWRI1* isoforms in *N. benthamiana* leaves.** **(A)** Schematic diagram of the binary vector constructs for the transient expression of *CsWRI1*s in *N. benthamiana* leaves. **(B)** Oil body (OB) counts in *N. benthaminana* leaves expressing empty vector (pBA002), *CsWRI1A. CsWRI1B*, or *CsWRI1C*. Values are averages and SD of three individual images. Data were statistically analyzed using Student’s *t-*test (^∗^*P* < 0.01). **(C)** *Agrobacterium* harboring *CsWRI1A. CsWRI1B*, or *CsWRI1C* was infiltrated in *N. benthamiana* leaves and then the leaf disks were stained with Nile red solution. The fluorescent signals were visualized by laser confocal scanning microscopy. The white arrows indicate OBs. Bars = 20 μm.

A limited number of *WRI1* genes identified from model plants, such as *Arabidopsis* for dicots (Cernac and Benning, 2004), *B. distachyon* for temperate grasses (Yang et al., 2015), and *B. napus* (Liu et al., 2010) and maize (Shen et al., 2010) for crops, have been tested for their ability to enhance the oil content in developing seeds and vegetative tissues (Grimberg et al., 2015). We tested the potential of each *Camelina* WRI1 isoform to enhance the vegetable oil biosynthesis in *N. benthamiana* leaves. Agrobacteria containing the plant expression vector cloned with each *CsWRI1* cDNA under the control of CaMV 35S promoter were infiltrated into *N. benthamiana* leaves (**Figure 8A**). The frequencies of oil bodies and fatty acid levels were examined and it was observed that leaf tissues expressing each of the CsWRI1 clearly indicated a greater number of oil bodies stained with Nile red than in the control leaves transformed with the empty vector (**Figures 8B,C**).

**FIGURE 9:**
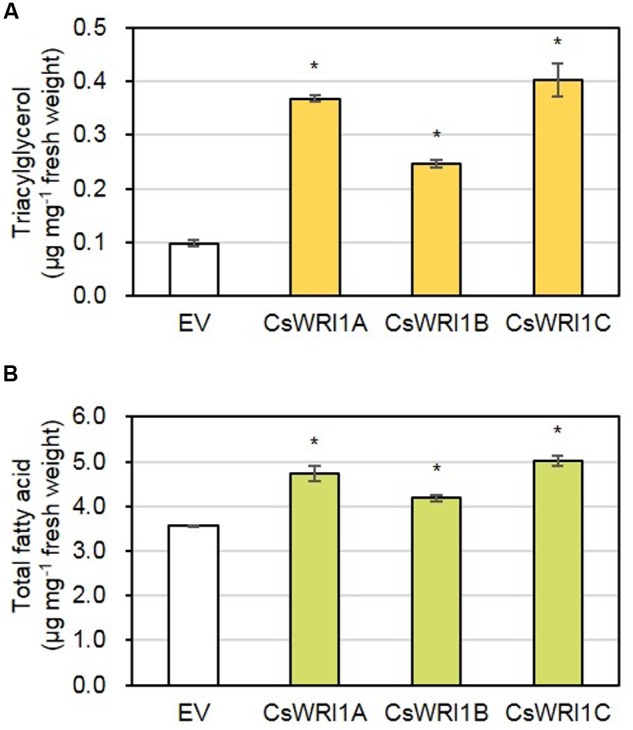
**Fatty acid content present in triacylglycerols** **(A)** and total lipids **(B)** of *N. benthamiana* leaves transiently overexpressing three CsWRI1 isoforms. *Agrobacterium* harboring empty vector (EV), *CsWRI1A. CsWRI1B*, or *CsWRI1C* was infiltrated to 5-week-old *N. benthamiana* leaves and the *N. benthamiana* plants were further incubated for 5 days. Total lipids were extracted from *N. benthamiana* leaf disks including the injection region and analyzed by gas chromatography or fractionated on thin layer chromatography. The eluted TAG fractions were transmethylated and the fatty acid methyl esters were analyzed by gas chromatography. Each value is the mean ± SE of three independent measurements. Data were statistically analyzed using Student’s *t*-test (^∗^*P* < 0.01).

In order to determine whether there is a correlation between the induced oil bodies in *N. benthamiana* leaves and an increased TAG content, *N. benthamiana* leaves infiltrated by *CsWRI1s* or the empty vector were measured for the fatty acid content in TAGs fractionated by TLC. Expression of each *CsWRI1* enhanced TAG content 2.5- to 4.0-fold in fresh leaves compared to the empty-vector control (**Figure 9A**). Measurement of the total lipid amount in fresh leaves showed that *CsWRI1s* produced 17–41% more fatty acids in transformed leaves compared with the control leaves transformed with the empty vector (**Figure 9B**). The TAG and total lipid content in leaves transformed by *CsWRI1s* had a fatty acid composition somewhat different to that of the controls. Five fatty acids (18:3 > 16:0 > 18:2 > 18:1 > 18:0) accounted for 97–98% of all TAGs. Transformation with *CsWRI1s* also induced a subtle change in the fatty acid composition of TAGs, such as an increase in 18:1 and a decrease in 18:0 compared to the empty vector controls. In the total lipid analysis, five fatty acids (18:3 > 16:0 > 18:2 > 16:3 > 18:0) accounted for 94–95% of all lipids. Among major fatty acids in leaves, 18:3 was decreased and 18:2 increased in transformed leaves compared to the control leaves (**Table 1**). The enhanced leaf TAG content suggests that CsWRI1s can be employed to increase the vegetable oil content in biomass.

**Table 1:** Fatty acid profile of the TAG fraction and total lipids of leaf disks transiently expressing the three *CsWRI1* genes.

	TAG fraction (mol %)				Total lipids (mol %)			
		
	EV	CsWRI1A	CsWRI1B	CsWRI1C	EV	CsWRI1A	CsWRI1B	CsWRI1C
16:0	25.2 ± 0.4	28.3 ± 0.2	26.5 ± 0.6	27.1 ± 0.3	19.4 ± 0.1	21.5 ± 0.3	22.5 ± 0.5	22.1 ± 0.4
16:1	1.3 ± 0.1	0.6 ± 0.0	0.7 ± 0.0	0.7 ± 0.0	2.7 ± 0.0	1.8 ± 0.1	2.1 ± 0.1	1.7 ± 0.2
16:3	1.0 ± 0.0	0.6 ± 0.0	0.5 ± 0.0	0.6 ± 0.0	7.8 ± 0.1	6.7 ± 0.2	5.9 ± 0.2	6.3 ± 0.1
18:0	6.1 ± 0.1	5.6 ± 0.0	5.3 ± 0.1	5.4 ± 0.0	3.1 ± 0.2	3.1 ± 0.3	3.2 ± 0.0	3.3 ± 0.2
18:1^Δ9+11^	5.1 ± 0.1	7.7 ± 0.2	8.9 ± 1.1	6.0 ± 1.0	1.4 ± 0.0	2.9 ± 0.2	3.0 ± 0.1	2.4 ± 0.4
18:2	23.5 ± 1.2	23.3 ± 0.3	26.2 ± 1.4	20.8 ± 1.6	10.4 ± 0.3	13.3 ± 0.5	14.0 ± 0.2	12.3 ± 0.7
18:3	35.9 ± 1.0	32.6 ± 0.4	30.7 ± 1.7	37.9 ± 2.2	54.8 ± 0.3	50.3 ± 1.1	48.9 ± 0.6	51.4 ± 1.5
20:0	1.1 ± 0.0	0.9 ± 0.0	0.8 ± 0.0	0.9 ± 0.0	0.4 ± 0.0	0.4 ± 0.0	0.5 ± 0.0	0.5 ± 0.0
22:0	0.4 ± 0.0	0.3 ± 0.0	0.3 ± 0.0	0.3 ± 0.0	-	-	-	-
24:0	0.3 ± 0.0	0.2 ± 0.0	0.1 ± 0.0	0.2 ± 0.0	-	-	-	-


*Error denotes standard error (SE) with *n* = 3 for all samples. EV is empty vector.*

## Discussion

*Camelina* has an advantage as an oil crop as it is amenable to metabolic engineering for the manipulation of vegetable oil biosynthesis (Bansal and Durrett, 2016). Metabolic engineering in *Camelina* is underway to modify the fatty acid composition by blocking the endogenous *fatty acid desaturase 2 (FAD)* and *fatty acid elongase (FAE) 1* pathways, which would make it more suitable for biofuel (Nguyen et al., 2013), and to incorporate new pathways to produce unusual fatty acids for industrial uses (Snapp et al., 2014; Liu et al., 2015; Nguyen et al., 2015), and omega-3 long-chain polyunsaturated fatty acids for human consumption (Petrie et al., 2014). Wax esters replaced 21% of the seed oil TAGs in *Camelina* transformed with two genes encoding a fatty alcohol-forming acyl-CoA reductase (FAR) and a wax ester synthase (WS) (Iven et al., 2016). Currently only two key genes that are involved in fatty acid and TAG biosynthesis metabolism, *FAD2* (Kang et al., 2011) and *DGAT1* (Kim et al., 2016), respectively, have been identified to exist in three copies in *Camelina*. In the present study, we show that the gene for *WRI1*, a regulator for fatty acid biosynthesis, also exists in three copies in the *Camelina* genome (**Figure 1**, **Supplementary Table 2**). The three copies (CsWRI1A, CsWRI1B, and CsWRI1C) were very similar in their amino acid sequences to those of *A. thaliana* (**Figure 1**). Phylogenetic tree analysis grouped the three CsWRI1s and those of *Arabidopsis* and Brassica to same clade (**Figure 2**). The AP2/EREBP DNA binding domain and a key ‘VYL’ motif for controlling the target gene’s transcription involved in fatty acid biosynthesis (Ma et al., 2013) were identically conserved but the N- and C-terminal amino acid sequences were moderately diverse in three *Camelina* and *Arabidopsis* WRI1s (**Figure 1**). A very high homology was observed among the three *Camelina WRI1* genes and *Arabidopsis WRI1*, which may be construed as evidence for a whole-genome triplication event from the ancestral crucifer genome (Kagale et al., 2014). The nuclear localization of WRI1 has not been confirmed in any previous report, although it has been shown that WRI1 binds to the regulatory element of the promoters in fatty acid biosynthesis as a transcription factor (Baud et al., 2009; Maeo et al., 2009). In this report, we demonstrated that WRI1s are localized in the nucleus by using fluorescent fusion proteins (**Figure 4**).

All three *Camelina* WRI1s were abundantly expressed in developing seeds (**Figure 3**). *CsWRI1A*, the gene with the highest homology with *Arabidopsis WRI1* exhibited higher expression levels than those of *CsWRI1B* and *CsWRI1C* (**Figure 3**). Ectopic expression of *Camelina* WRI1s partially replaced *Arabidopsis* WRI1 functionality by partially recovering the reduced amount of fatty acids and reversing the wrinkled phenotype in *wri1-3* seeds as compared to those of the wild-type (**Figures 5** and **6**). Each of *CsWRI1*s transgenic lines upregulated *PI-PKβ1* and *BCCP2* downstream target genes in developing seeds compared with *wri1-3* host plants (**Figure 7**). The reason that CsWRI1s could not fully recover the fatty acid amount and reverse the wrinkled phenotype in transgenic seeds may be that the use of the CaMV 35S promoter or the regulation of CsWRI1 by endogenous factors FUS3 and LEC2 may not be optimal for seed oil accumulation (Devic and Roscoe, 2016). The expression of *phosphatidylcholine diacylglycerol cholinephosphotransferase* (*PDCT*) of flax (*Linum usitatissimum*) under the control of CaMV 35S promoter in *Arabidopsis rod1* mutant could not recover the PUFA content comparable with that observed in wild-type plants (Wickramarathna et al., 2015). In addition, the introduction of a vector combined with CaMV 35S promoter and *B. rapa FAD2-1* gene into *Arabidopsis fad2-2* loss-of-function mutant did not achieve the level of linoleic acid found in seeds of the wild-type plant (Jung et al., 2011). On the other hand, a recent study showed that the overexpression of WRI1 by *FUS3* seed-specific promoter resulted in a stronger effect than that from CaMV 35S promoter alone for the induction of more oil production in seeds (Kanai et al., 2016). These similar results suggest that the CaMV 35S promoter might not be appropriate for metabolic engineering to enhance seed oil accumulation.

Oil production in leaves is an alternative way to increase the production of vegetable oils in limited cultivating lands and under water supply constraints to meet the increasing demand for vegetable oils as foods and biofuels. Oil accumulation in developing seed requires seed maturation controlled by four master regulators: ABI3, FUS3, LEC1, and LEC2. LEC2 is expressed early on in seed development, while FUS3 and ABI3 are more implicated in seed maturation processes (Wang et al., 2007; Wang and Perry, 2013; Roscoe et al., 2015; Zhang et al., 2016). WRI1 is a regulator of lipid accumulation downstream of the above four master regulators (Roscoe et al., 2015). Recently, researchers have attempted to produce vegetable oil (TAG or fatty acids) in leaves with the overexpression of a key regulator *WRI1* for fatty acid synthesis as well as senescence-inducible or xylem-specific expression of *LEC2*, which is a transcription factor involved in the early stages of seed development (Kim et al., 2015). In most cases, since *Arabidopsis WRI1* gene has not shown any negative effect on plant growth, it has been used mainly for the production of TAG in the vegetative tissues. Overexpression of *Arabidopsis WRI1* alone elevated TAG levels to 2.8-fold in its seedlings (Sanjaya et al., 2011). Co-expression of *Arabidopsis WRI1* and *DGAT1*, involved in the final step of TAG biosynthesis, and oleosin, responsible for the stability of TAG, resulted in an accumulation of 15% TAG of the estimated 17.7% total lipids in *N. benthamiana* leaves (Vanhercke et al., 2014). Co-expression of *WRI1* and *DGAT1-2* in a transgenic sugarcane plant, which also possessed suppressed endogenous genes for ADP-glucose pyrophosphorylase (AGPase) and peroximal ABC transport 1 (PXA1), resulted in increased total fatty acid content of up to 4.7 and 1.7% in leaves and stems, respectively (Zale et al., 2016). Since WRI1 is a regulator for lipid accumulation even in non-seed tissues (Kilaru et al., 2015), WRI1 paralogs distinct from seed-specific WRI1 were observed in the tallow layer, the non-seed tissue, in Chinese tallow (*Triadica sebifera*), which is a valuable oilseed-producing tree (Divi et al., 2016). In yet another study, the ectopic expression of *B. distachyon* WRI1 isolated from the vegetative tissues induced an increase in TAG content and simultaneously resulted in cell death in the leaf blades (Yang et al., 2015). Ectopic expression of *WRI1*s from diverse species and tissues in leaves of *N. benthamiana* has been shown to result in the production of TAG content ranging from 0.05 to 2.2%. (Grimberg et al., 2015). In the present study, the overexpression of three *Camelina WRI1* isoforms resulted in the formation of oil bodies (**Figure 8**) and the production of approximately 0.025–0.04% TAG in fresh leaves, which represents a 2.5- to 4.0-fold increase from control leaves (**Figure 9A**). The amount of total lipids in fresh leaves was increased by a further 17–41% by introducing *CsWRI1s* (**Figure 9B**). The fatty acid composition of TAG and leaves was also found to be different (**Table 1**). Two major fatty acid levels were altered: 18:1 was increased and 18:0 was decreased in the TAG fraction, whereas 18:3 was decreased and 18:2 was increased in the total lipids of *CsWRI1s* transformants, compared with empty vector control leaves. This suggests CsWRI1s may drive 18:1 deposition for TAG synthesis. In conclusion, three *WRI1* genes isolated from the hexaploid genome of *Camelina*, an emerging oil seed crop, can be used for the regulation of transcriptional factors to produce vegetable oils in engineered biomass.

## Author Contributions

MCS and HUK designed the research; DA, HK, SJ, and YSG did experiments; and DA, HK, HUK, and MCS analyzed the data and wrote a manuscript.

## Conflict of Interest Statement

The authors declare that the research was conducted in the absence of any commercial or financial relationships that could be construed as a potential conflict of interest.

## Footnotes

1 http://www.megasoftware.net/

2 http://camelinagenomics.org/

3 http://www.camelinadb.ca/
