# Supplementary material for: Expression of Camelina WRINKLED1 Isoforms Rescue the Seed Phenotype of the Arabidopsis wri1 Mutant and Increase the Triacylglycerol Content in Tobacco Leaves
Source: Front Plant Sci. 2017 Jan 24;8:34. doi: 10.3389/fpls.2017.00034 (PMC5258696; doi:10.3389/fpls.2017.00034)
Supplement: Supplementary file 1 [file Presentation_1.PDF]

A

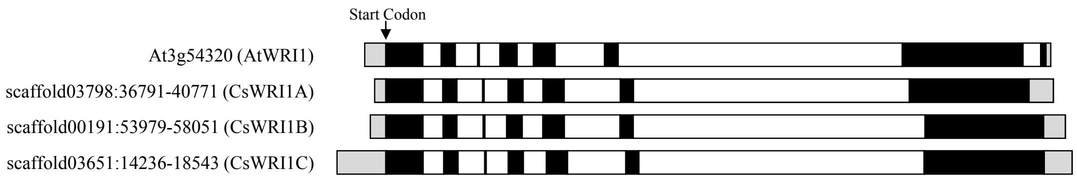

B

Query sequence	Query length (bp)			
	Genomic DNA (including UTR, Intron and Exon sequence)	5'UTR	3'UTR	Intron
AtWRI1	3,944	125	28	2,474
scaffold03798:36791-40771 (CsWRI1A)	3,981	69	143	2,467
scaffold00191:53979-58051 (CsWRI1B)	4,073	94	128	2,555
scaffold03651:14236-18543 (CsWRI1C)	4,308	289	164	2,550

C

Query Coverage (%)

Identity (%)

Query sequence	5'UTR				3'UTR				Intron			
	AtWRI1	CsWRI1A	CsWRI1B	CsWRI1C	AtWRI1	CsWRI1A	CsWRI1B	CsWRI1C	AtWRI1	CsWRI1A	CsWRI1B	CsWRI1C
AtWRI1		33	24	82		53	53	64		95	81	94
CsWRI1A	93		92	88	93		98	98	71		100	100
CsWRI1B	100	90		98	93	90		100	75	84		100
CsWRI1C	84	95	91		94	87	97		70	85	91	

Supplementary Fig. 1 Structural organization (A and B) and BLAST analysis of identified UTR and intron in *WRI1* gene from *A. thaliana* and *C. sativa*. (A) Schematic representation of the *WRI1* gene models of *A. thaliana* and *C. sativa*. Black boxes indicate the protein-coding regions of exons. Open white boxes represent introns. Grey boxes represent the 5′- or 3′-UTR. (B) The length of sequences in the genomic DNA, 5′- and 3′-UTR, and the intron region. (C) BLAST analysis of UTR and intron sequences using blastn program (https://blast.ncbi.nlm.nih.gov).

```
CsWRI1A                 1 ATGAAGAGGCGCTTAACGAGTACGAATTCCTCTTCTTCTTCTCCATCTTCCTCTGTTTCTTCTTCTACTACTACTTCCTCACCTATTCAA
CsWRI1B                 1 ATGAAGAGGCGCTTAACGAGTACGAATTCTTCTTCTTCTTCTCCATCTTCCTCTGTTTCTTCTTCAACTACTACTTCATCTCCTATTCAA
CsWRI1C                 1 ATGAAGAGGCGCTTAACGAGTACGAATTCTTCTTCTTCTTCTCCATCTTCCTCTGTTTCTTCTTCAACTACTACTTCATCTCCTATTCAA
AtWRI1_NM_001035780     1 ATGAAGAAGCGCTTAACCACTTCCA---CTTGTTCTTCTTCTCCATCTTCCTCTGTTTCTTCTTCTACTACTACTTCCTCTCCTATTCAG

CsWRI1A                91 TCGGAGACTCCACGGCCTAAACGAGCCAACAAGAGGGCTAAGAAGCCTTCTCCTTCTGATGATAAAACGACTAACCCGACAAGCCCTGCT
CsWRI1B                91 TCGGAGACTCCACGGCCTAAACGAGCCAACAAGAGGGCTAAGAAGCCTTCTCCTTCTGGTGATAAACCAAATAACCCGACAAGCCCTGCT
CsWRI1C                91 TCGGAGACTCCACGGCCTAAACGAGCCAACAAGAGGGCTAAGAAGCCTTCTCCTTCTGATGACAAACCAAATAACCCGACAAGCCCTGCT
AtWRI1_NM_001035780    88 TCGGAGGCTCCAAGGCCTAAACGAGCC---AAAAGGGCTAAGAAATCTTCTCCTTCTGGTGATAAATCTCATAACCCGACAAGCCCTGCT

CsWRI1A               181 TCCACCAGACGCAGCTCTATCTACAGAGGAGTCACTAGACATAGATGGACTGGGAGATTCGAGGCTCATCTTTGGGACAAAAGCTCTTGG
CsWRI1B               181 TCCACCAGACGCAGCTCTATCTACAGAGGAGTCACTAGACATAGATGGACTGGGAGATTCGAGGCTCATCTTTGGGACAAAAGCTCTTGG
CsWRI1C               181 TCCACCAGACGCAGCTCTATCTACAGAGGAGTCACTAGGCATAGATGGACTGGGAGATTCGAGGCTCATCTTTGGGACAAAAGCTCTTGG
AtWRI1_NM_001035780   175 TCTACCCGACGCAGCTCTATCTACAGAGGAGTCACTAGACATAGATGGACTGGGAGATTCGAGGCTCATCTTTGGGACAAAAGCTCTTGG

CsWRI1A               271 AATTCGATTCAGAACAAGAAAGGCAAACAAGTTTATCTGGGAGCATATGACAGCGAAGAAGCAGCAGCACATACGTACGATCTGGCTGCT
CsWRI1B               271 AATTCGATTCAGAACAAGAAAGGCAAACAAGTTTATCTGGGAGCATATGACAGCGAAGAAGCAGCAGCACATACGTACGATCTGGCTGCT
CsWRI1C               271 AATTCGATTCAGAACAAGAAAGGCAAACAAGTTTATCTGGGAGCATATGACAGCGAAGAAGCAGCAGCACATACGTACGATCTGGCTGCT
AtWRI1_NM_001035780   265 AATTCGATTCAGAACAAGAAAGGCAAACAAGTTTATCTGGGAGCATATGACAGTGAAGAAGCAGCAGCACATACGTACGATCTGGCTGCT

CsWRI1A               361 CTCAAGTACTGGGGACCCGATACCATCTTGAATTTTCCGGCAGAGACGTATACAAAGGAGTTGGAGGAAATGCAGAGAGTGACAAAGGAA
CsWRI1B               361 CTCAAGTACTGGGGACCCGACACCATCTTGAATTTTCCGGCAGAGACGTATACAAAGGAGTTGGAAGAAATGCAGAGAGTGACAAAGGAA
CsWRI1C               361 CTCAAGTACTGGGGACCCGACACCATCTTGAATTTTCCGGCAGAGACGTATACAAAGGAGTTGGAAGAAATGCAGAGAGTGACAAAGGAA
AtWRI1_NM_001035780   355 CTCAAGTACTGGGGACCCGACACCATCTTGAATTTTCCGGCAGAGACGTACACAAAGGAATTGGAAGAAATGCAGAGAGTGACAAAGGAA

CsWRI1A               451 GAATATTTGGCTTCTCTCCGCCGCCAGAGCAGTGGTTTCTCTAGAGGCGTCTCTAAATATCGCGGCGTCGCTAGGCATCACCATAACGGA
CsWRI1B               451 GAATATTTGGCTTCTCTCCGCCGCCAGAGCAGTGGTTTCTCTAGAGGCGTCTCTAAATATCGCGGCGTCGCTAGGCATCACCATAACGGA
CsWRI1C               451 GAATATTTGGCTTCTCTCCGCCGCCAGAGCAGTGGTTTCTCTAGAGGCGTCTCTAAATATCGCGGCGTCGCTAGGCATCACCATAACGGA
AtWRI1_NM_001035780   445 GAATATTTGGCTTCTCTCCGCCGCCAGAGCAGTGGTTTCTCCAGAGGCGTCTCTAAATATCGCGGCGTCGCTAGGCATCACCACAACGGA

CsWRI1A               541 AGATGGGAGGCTCGGATTGGAAGAGTGTTTGGAAACAAGTACTTGTACCTCGGCACCTACAATACGCAGGAGGAAGCTGCAGCGGCTTAT
CsWRI1B               541 AGATGGGAGGCTCGGATTGGAAGAGTTTTTGGAAACAAGTACTTGTACCTCGGCACCTACAATACGCAGGAGGAAGCTGCAGCGGCTTAT
CsWRI1C               541 AGATGGGAGGCTCGGATTGGAAGAGTGTTTGGAAACAAGTACTTGTACCTCGGCACCTACAATACGCAGGAGGAAGCTGCAGCGGCTTAT
AtWRI1_NM_001035780   535 AGATGGGAGGCTCGGATCGGAAGAGTGTTTGGGAACAAGTACTTGTACCTCGGCACCTATAATACGCAGGAGGAAGCTGCTGCAGCATAT

CsWRI1A               631 GACATGGCAGCCATAGAGTATCGAGGTGCCAATGCGGTTACTAATTTCGACATTAGTAATTACATCGACCGGTTAAAGAAGAAAGGTGTT
CsWRI1B               631 GACATGGCAGCTATAGAGTATCGAGGTGCCAACGCGGTTACTAATTTCGACATTAGTAATTACATCGACCGGTTAAAGAAGAAAGGTGTT
CsWRI1C               631 GACATGGCAGCTATAGAGTATCGAGGTGCCAACGCGGTTACTAATTTCGACATTAGTAATTACATCGACCGCTTAAAGAAGAAAGGCGTT
AtWRI1_NM_001035780   625 GACATGGCTGCGATTGAGTATCGAGGCGCAAACGCGGTTACTAATTTCGACATTAGTAATTACATTGACCGGTTAAAGAAGAAAGGTGTT

CsWRI1A               721 TTCCCGTTCCCTGTGAGCCAAGCTAACCATCAAGAAGAGGCTATACTTGCTGAAGCCAAACAAGAGATTGAA---------GCGAAAGAA
CsWRI1B               721 TACCCGTTCCCTGTGAGCCAAGCTAACCATCAAGAAGAGGCTATTCTTGCTGAAGCCAAACAAGAGATTGAA---------GCTAAAGAA
CsWRI1C               721 TTCCCGTTCCCTGTGAGCCAAGCTAACCATCAAGAAGAGGCTATACTTGCTGAAGCCAAACAAGAGATTGAA---------GCTAAAGAA
AtWRI1_NM_001035780   715 TTCCCGTTCCCTGTGAACCAAGCTAACCATCAAG---AGGGTATTCTTGTTGAAGCCAAACAAGAAGTTGAAACGAGAGAAGCGAAGGAA

CsWRI1A               802 GAGCCTACAGAAGAAGTGAAACAACAGTGCGTGGAAGAGCCACCACAA---------GTACAGAAAGAAGAGAAAGCAGAGCAGCAAGGA
CsWRI1B               802 GAACCTACAGAAGAAGTGAAACAACAGTACGTGGAAGAGCCACCACAACAA------GTACAGAAAGAAGAGAAAGCAGAGCAGCAAGGA
CsWRI1C               802 GAGCCTACAGAAGAAGTGAAACAACAGTGCGTGGAAGAGCCACCACAA---------GTACAGAAAGAAGAGAAAGCAGAGCAGCAAGGA
AtWRI1_NM_001035780   802 GAGCCTAGAGAAGAAGTGAAACAACAGTACGTGGAAGAACCACCGCAAGAAGAAGAAGAGAAGGAAGAAGAGAAAGCAGAGCAACAAGAA

CsWRI1A               883 GAAGAGGTTGTGGGATATAA---AGAAGAAGAGGCGGCGGTGGTCAATTGCTGCATAGACTCTTCAGCCATAATGGAAATGAATCGTTGT
CsWRI1B               886 GAAGAGTTTGTGGCATACAA---AGAAGAAGAGGCGGCGGTGGTCAATTGCTGCATAGACTCTTCAGCCATAATGGGAATGAATCGTTGT
CsWRI1C               883 GAAGAGTTTGTGGGATATAATAAAGAAGAAGAGGCGACGGTGGTCAATTGCTCCATAGACTCTTCAGCCATAATGGAAATGAATCGTTGT
AtWRI1_NM_001035780   892 GCAGAGATTGTAGGATATTC------AGAAGAAGCAGCAGTGGTCAATTGCTGCATAGACTCTTCAACCATAATGGAAATGGATCGTTGT

CsWRI1A               970 TCGAATGACAACGAGCTGGCTTGGAACTTCTGTATGATGGATTCAGGGTTTACTCCGTTCTTGACGGATCAGAATCTCTCAAATGAGAAT
CsWRI1B               973 TCGGACGACAATGAGCTGGCTTGGAACTTCTGTATGATGGATTCAGGGTTTGCTCCGTTCTTGACGGATCAAAATCTCCCTAATGAGAAT
CsWRI1C               973 TCGGACGACAATGAGCTGGCTTGGAACTTCTGTATGATGGATTCAGGGTTTGCTCCCTTCTTGACGGATCAGAATCTCTCAAATGAGAAT
AtWRI1_NM_001035780   976 GGGGACAACAATGAGCTGGCTTGGAACTTCTGTATGATGGATACAGGGTTTTCTCCGTTTTTGACTGATCAGAATCTCGCGAATGAGAAT

CsWRI1A              1060 CCTATCGAGTATCCAGAGCTTTTCAATGAGTTAGCATTTGAGGAGAACATTGACTTCATGTTCGAGGAAGGGAAGAACGAGTGCTTGGGG
CsWRI1B              1063 CCCATCGAGTATCCCGAGCTTTTCAACGAGTTAGCATTTGAGGAGAACATTGACTTCATGTTCGAGGAAGGGAAGAACGAGTACTTGGGG
CsWRI1C              1063 CCCATCGAGTATCCAGAGCTTTTCAACGAGTTAGCATTTGAGGAGAACATTGACTTCATGTTCGAGGAAGGGAAGAACGAGTGCTTGGGG
AtWRI1_NM_001035780  1066 CCCATAGAGTATCCGGAGCTATTCAATGAGTTAGCATTTGAGGACAACATCGACTTCATGTTCGATGATGGGAAGCACGAGTGCTTGAAC

CsWRI1A              1150 TTGGGAAATCTGGATTGTTGCGAGGTT---GTGGTGGGAAGAGAGAGCCCAACTTCTTCGTCTTCACCTTTGTCTTGCTTTTCTACCGAC
CsWRI1B              1153 TTGGGAAATCTGGATTGTTGCGATGTTGTTGTGGTGGGAAGAGAGAGCCCAACTTCTTCGTCTTCTCCGTTGTCTTGCTTTTCTACTGAC
CsWRI1C              1153 TTGGGAAATATGGATTGTTGCGATGTTGTTGTGGTGGGAAGAGAGAGCCCAACTTCTTCGTCTTCTCCGTTGTCTTGCTTTTCTACTGAC
AtWRI1_NM_001035780  1156 TTGGAAAATCTGGATTGTTGCG---------TGGTGGGAAGAGAGAGCCCACCCTCTTCTTCTTCACCATTGTCTTGCTTATCTACTGAC

CsWRI1A              1237 TCTGCTTCATCAACGACAACAACAACAGCAACAACAACCTCTGTTTCTTGTAACTATTCTGTCTGA
CsWRI1B              1243 TCTGCTTCATCAT------------CAACAACAACAACCTCTGTTTCTTGTAACTATTCTGTCTGA
CsWRI1C              1243 TCTGCTTCATCAAC---AACAACAGCAACAACAACAACCTCTGTTTCTTGTAACTATTCTGTCTGA
AtWRI1_NM_001035780  1237 TCTGCTTCATCAAC---------AACAACAACAACAACCTCGGTTTCTTGTAACTATTTGGTCTGA
```

Supplementary Fig. 2 Alignment of the nucleotide sequences of *WRI1* isoforms from *C. sativa* and *Arabidopsis*. Non-conserved and conservatively changed amino acid residues are shaded in black and grey, respectively.
